# Race, oxygen exposure, and retinopathy of prematurity: re-examining a persistent epidemiologic paradox

**DOI:** 10.3389/ebm.2026.11094

**Published:** 2026-05-18

**Authors:** Beryl Zhou, Sarah H. Rodriguez, Alexis Warren, Dimitra Skondra

**Affiliations:** 1 Pritzker School of Medicine, University of Chicago, Chicago, IL, United States; 2 Department of Ophthalmology and Visual Science, University of Chicago, Chicago, IL, United States; 3 Department of Ophthalmology, NYU Grossman School of Medicine, New York, NY, United States

**Keywords:** oxygen exposure, race, retinopathy of prematurity, socioeconomic determinants of health, survival analysis

## Abstract

Retinopathy of prematurity (ROP) is a leading cause of childhood blindness that arises from disrupted retinal vascular development in premature infants. Oxygen exposure remains a central driver of treatment-warranted ROP, as higher saturation levels suppress early retinal vascular growth and later promote pathological neovascularization. Large, randomized trials of oxygen targeting show that lower oxygen saturation ranges reduce the incidence of treatment-requiring ROP, though with trade-offs in mortality. Observational cohorts, including the CRYO-ROP, ETROP, and e-ROP trials, consistently report lower rates of treatment-warranted ROP and reduced treatment need among Black infants despite similar or greater prematurity risk. Multiple explanations have been proposed to account for the paradoxically lower rates of treatment-warranted ROP observed among Black infants. Although biologic variations in angiogenic pathways have been proposed, evidence suggests that structural and clinical factors may offer an alternative explanation for these patterns. Black race is strongly correlated with residence in neighborhoods with greater socioeconomic disadvantage, which is associated with increased risk of prematurity and missed ROP follow-up visits. In addition, pulse oximeters may overestimate oxygen saturation in individuals with darker skin pigmentation, potentially altering targeted oxygen exposure. Survival-related selection bias may further contribute to this paradox, as infants at the highest risk of both mortality and treatment-warranted ROP may not survive long enough to develop treatment-requiring disease. This review examines racial differences in ROP severity and examines how oxygen exposure and structural factors may contribute to these disparities, while acknowledging the limited evidence supporting biologic explanations.

## Impact statement

For decades, studies have suggested that Black infants develop treatment-warranted retinopathy of prematurity (ROP) less often than White infants, creating a persistent and incompletely understood paradox. This review re-examines that observation by integrating evidence from epidemiology, neonatal care, and social determinants of health. Beyond merely a biological protective effect, we highlight how differences in survival patterns, access to care, and oxygen monitoring practices may influence the observed rates of severe disease. By synthesizing evidence across these domains, this work reframes how racial differences in ROP should be interpreted. The manuscript advances the field by shifting the focus from presumed biological differences toward structural and clinical factors, while providing a framework for future research that can improve care for premature infants.

## Introduction

Retinopathy of prematurity (ROP) is a vaso-proliferative retinal disorder and a leading cause of childhood blindness [1]. The disease arises from disruption of normal retinal vascular development, driven primarily by prematurity and postnatal environmental factors. Following the first epidemic of ROP in the 1940s and 1950s due to unrestricted oxygen use, oxygen toxicity has been recognized as a central contributor to abnormal retinal vascular development [2, 3]. ROP ranges across several stages of severity, with some requiring laser or injections for treatment. As this review pertains to studies for which treatment criteria have changed over time, threshold ROP (as defined by CRYO-ROP) and Type 1 ROP (as defined by ET-ROP) are both referred to as “treatment-warranted ROP” throughout [4, 5].

**FIGURE 1:**
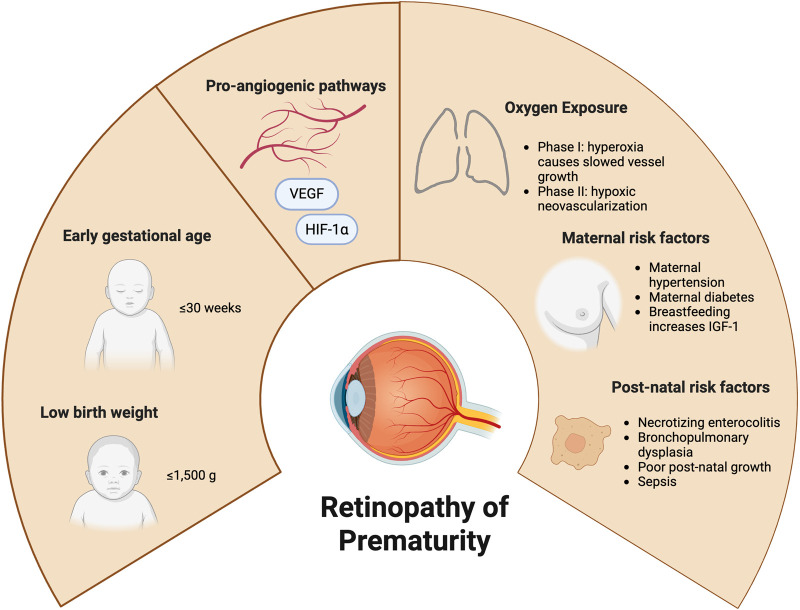
Risk factors for retinopathy of prematurity, including low birth weight, early gestational age, oxygen exposure, and components of maternal and post-natal health. Many of these are mediated by pro-angiogenic pathways, such as vascular endothelial growth factors (VEGF) and hypoxia-inducible factor (HIF-1a). Images created in BioRender.com.

Major risk factors for ROP include low gestational age (GA) and birth weight (BW), with current US guidelines recommending screening for all infants ≤30 weeks of GA or ≤1,500 g of BW [6]. Maternal risk factors include hypertensive disorders of pregnancy, diabetes, medication exposures, smoking, and advanced maternal age. Neonatal factors include prolonged mechanical ventilation (>7 days), bronchopulmonary dysplasia (BPD), impaired erythropoiesis, thrombocytopenia, patent ductus arteriosus, bacterial and fungal sepsis, poor postnatal growth, and necrotizing enterocolitis [7–11]. Nutritional factors such as breast milk intake and supplementation with fish oil containing omega-3 fatty acids may also be protective (Figure 1) [12–14].

Despite advances in oxygen management and neonatal care, epidemiologic studies have repeatedly suggested that race and ethnicity may influence the risk of ROP. The multicenter study of cryotherapy for ROP (CRYO-ROP) showed a lower incidence of threshold ROP in Black infants than White infants in North American populations, even after adjusting for BW and GA [4]. Subsequent cohort studies and neonatal databases have reported similar findings, suggesting a relative reduction in treatment-warranted ROP among Black infants [15–18]. However, these observations are inconsistent and remain mechanistically unexplained. Some studies have similarly suggested a protective association of Black race [11, 19, 20], whereas others report no difference or conflicting findings [11, 21]. The relationship between race, oxygen exposure, and ROP therefore represents an unresolved epidemiological pattern. This review examines racial differences in ROP and evaluates biologic, clinical, and socioeconomic mechanisms that may contribute to these observations.

### Oxygen exposure and ROP pathogenesis

ROP develops through a biphasic model driven by oxygen-mediated disruption of retinal angiogenic signaling [3]. *In utero*, the developing retina typically exists in a relatively hypoxic environment that maintains hypoxia-inducible factor (HIF) and vascular endothelial growth factor (VEGF) expression at normal levels to support organized vascularization. Following premature birth, exposure to higher oxygen tension suppresses HIF signaling and reduces VEGF expression, resulting in slowed vascular growth (phase I) [22]. Low insulin-like growth factor 1 (IGF-1) levels further disrupts physiologic vessel growth, due to limited nutritional and hepatic production [23].

As the peripheral retina matures, metabolic demand increases despite incomplete vascularization. The resulting tissue hypoxia drives further upregulation of VEGF, erythropoietin (EPO), and other pro-angiogenic mediations, leading to disorganized vessel formation (phase II). As the infant matures, IGF-1 levels rapidly rise to stimulate abnormal neovascularization in Phase II [24]. Because these angiogenic pathways are highly-oxygen sensitive, even modest differences in oxygen exposure may alter disease severity.

Landmark clinical trials have demonstrated this relationship (Table 1). The Supplemental Therapeutic Oxygen for Prethreshold ROP (STOP-ROP) study found that higher oxygen supplementation did not reduce progression to ROP and revealed increased adverse pulmonary complications [25]. Notably, a *post hoc* subgroup analysis found that increasing supplemental oxygen in infants without plus disease during Phase II could decrease the progression to threshold disease. Similarly, large multicenter trials such as Surfactant, Positive Airway Pressure, and Pulse Oximetry Trial (SUPPORT), BOOST, and the Canadian Oxygen Trial (COT) investigated different oxygen-saturation targets in extremely preterm infants [26–28]. Across trials, restriction of oxygen within the target range of 85–89% reduced treatment-warranted ROP, but were associated with increased morbidity and mortality [29]. In contrast, higher targets within the 91–96% oxygen-saturation range were associated with increased odds of treatment-warranted ROP [30]. The Neonatal Oxygenation Prospective Meta-analysis Collaboration (NeOProM) analysis from 5 randomized clinical trials reported that lower oxygen saturation targets reduced the likelihood of requiring treatment for ROP, but also increased NEC and mortality (with one additional death for every 2 cases of treatment-warranted ROP avoided) [31–33]. These studies demonstrate complex trade-offs between survival and ROP risk, reinforcing that oxygen exposure remains a key modifiable determinant of pathogenesis.

**TABLE 1:** Landmark clinical studies described in this review.

Study	Design	Methods	Results & key takeaways
STOP-ROP [24]	Randomized controlled trial	325 pre-threshold infants at oxygen targets of 89–94% 324 pre-threshold infants at oxygen targets of 96–99%	Higher oxygen supplementation did not reduce or cause additional progression to TW-ROP. Increased adverse pulmonary complications (pneumonia, chronic lung disease) occurred in infants in higher oxygen targets More infants in the higher oxygen saturation group were still on supplemental oxygen in hospital at 3 months
SUPPORT [25]	Controlled multicenter trial with 2-by-2 factorial design	654 infants at oxygen targets of 85–89% 662 infants at oxygen targets of 91–95%	Lower target ranges reduced risk of TW-ROP among survivors but was associated with an increased risk of mortality prior to discharge No significant decrease in the composite outcome of severe retinopathy or death Rate of oxygen use at 36 weeks was reduced in the lower-oxygen saturation group
BOOST II [26]	​	934 infants at oxygen targets of 85–89%; 723 infants at oxygen targets of 91–95%	Post-hoc combined analyses showed significantly increased risk of death alone in lower-target groups compared to higher target groups (RR, 1.45; 95% CI, 1.16 to 1.82; P = 0.001) at 2 years More infants were treated for ROP in the higher-target group, but no significant difference was found between-groups in the rate of blindness
COT [27] (2013)	Randomized controlled trial	602 infants at oxygen targets of 85–89%; 599 infants at oxygen targets of 91–95%	The rate of death or disability was not significantly higher in the lower-target group than the higher-target group (RR 1.08; 95% CI, 0.85–1.37; p = 0.52) at 18 months. No significant difference found on ROP incidence and BPD. Lower targets compared with higher targets reduced the mean PMA at the last oxygen therapy from 36.2 to 35.4 weeks (p = 0.03)
CRYO-ROP [4]	Prospective cohort study	2,158 white infants and 1,568 black infants	ROP incidence was similar across all racial subgroups, but severe threshold ROP was less common in low-birth-weight black infants
ET-ROP [5]	Retrospective re-analysis of the ET-ROP data [28]	401 randomized patients	Retrospective re-analysis of the ET-ROP data (2014)* found that african american race (n = 64) had a significantly higher risk of developing zone I ROP (OR = 3.78, CI 2.06–6.96, p < 0.001) compared to caucasian patients (n = 230) [28].
NeOProM [29]	Prospective meta-analysis from 5 RCTs	2,480 infants at oxygen targets of 85–89%; 2,485 infants at oxygen targets of 91–95%	Lower oxygen saturation targets reduced the likelihood of requiring treatment for ROP (10.9% vs. 14.9%) but also increased NEC and mortality (with one additional death for every 2 cases of treatment-warranted ROP avoided)
e-ROP [16]	Multicenter observational cohort study	979 infants without referral-warranted ROP at first eye examination	GA, BW, nonblack race, and slow weight gain were associated with an increased risk of developing referral-warranted ROP.
G-ROP-1 (2015–2017) G-ROP-2 (2006–2011)	Secondary retrospective analysis of the multicenter studies (2023) [11]	White (N = 5,580), black (N = 3,252), and asian (N = 353)	Black infants had significantly lower BW and GAs compared with white and asian infants yet also demonstrated lower adjusted risks of severe ROP (11.1% vs. 12.4% vs. 11.9%), any stage ROP (42.1% vs. 43.2% vs. 30.6%), and plus disease (3.6% vs. 6.3%, vs. 5.9%) Mean daily-weight-gain were similar across groups on days of life 11–20 and 21–30, but lower in black and asian infants on days 31–40 (p < 0.001)

STOP-ROP = supplemental therapeutic oxygen for prethreshold retinopathy of prematurity; SUPPORT = surfactant, Positive Pressure, and Oxygenation Randomized Trial; BOOST = benefits of oxygen saturation targeting; COT = canadian oxygen trials; CRYO-ROP = cryotherapy for retinopathy of prematurity; ET-ROP = early treatment for retinopathy of prematurity; NeOPROM: Neonatal Oxygenation Prospective Meta-Analysis Collaboration; e-ROP: Evaluating Acute-Phase Retinopathy of Prematurity; G-ROP = postnatal growth and retinopathy of prematurity study.

TW-ROP = treatment-warranted retinopathy of prematurity; GA = gestational age; BW = birth weight; OR = odds ratio; RR = relative risk; CI = confidence interval; NEC = necrotizing enterocolitis.

* abstract only.

### Racial differences and ROP severity

Observational studies spanning several decades suggest that race may influence treatment-warranted ROP risk, although the mechanisms underlying this association remain unclear. The initial CRYO-ROP study reported lower treatment-warranted ROP incidence in low-birth-weight Black infants despite similar overall incidence of ROP compared to White infants [4]. Subsequent studies including the Early Treatment for Retinopathy of Prematurity (ETROP) and the Evaluating Acute-Phase Retinopathy of Prematurity (e-ROP) demonstrated similar findings, with Black race associated with lower treatment-warranted disease compared to non-Black infants despite overall comparable ROP incidence [15, 16].

Additional cohort studies by Yang et al. and Port et al. further supported these findings by identifying Black race to be an independent protective factor for treatment-requiring ROP in multivariable analyses [17, 18]. In the Postnatal Growth and ROP (G-ROP-1 and G-ROP-2) studies, Black infants had significantly lower BW and GAs compared with White and Asian infants, yet also demonstrated lower adjusted risks of treatment-warranted ROP, any stage ROP, and plus disease [11]. Importantly, mean daily weight gain were similar during the first 30 days of life, and there were no differences in the timing of ROP onset or progression across racial groups. At days 31–40, however, postnatal daily-weight-gain was significantly lower among Black infants compared to White infants. Additionally, they found that Black infants were less likely to develop Zone 1 disease, which contradicted with a secondary analysis of the ET-ROP database that showed the opposite [34]. These findings suggest that differences in postnatal growth or early disease kinetics may not be able to fully explain the observed differences.

Although these epidemiological observations have been replicated across multiple cohorts (Table 1), they must be interpreted cautiously. A Los Angeles cohort study reported that apparent race/ethnicity differences in ROP outcomes were mainly driven by GA and neighborhood income rather than race alone [35]. Black infants were more likely to be born at lower GAs than non-Hispanic White neonates, but this association was attenuated after adjustment for household income and insurance status. Lower household income remained significantly associated with earlier gestational age, highlighting the important role of socioeconomic determinants in shaping prematurity risk, and consequently, ROP susceptibility.

### Genetic susceptibility and biological mechanisms

Although structural factors provide important context, researchers have also explored whether biological mechanisms could contribute to racial differences in ROP severity. An early hypothesis by Saunders et al. proposed that retinal pigmentation might confer protection against oxidative injury, whereas Good et al. suggested that β-blocker receptor polymorphisms may play a role in their immunity to severe disease [4, 15]. Alternatively, Monos et al. speculated that greater amounts of melanin in the retinal pigment epithelium (RPE) or choroid would be a protective factor against developing ROP, independent of BW, GA, and length of oxygen therapy [36]. A subsequent study by Fan et al. found that light fundus pigmentation was consistently associated with higher risk features compared to medium or dark fundus pigmentation [37]. Melanin functions as a free radical scavenger to protect the retina from oxidative stress, providing a plausible mechanism for reducing ROP progression in high-risk infants [38]. In other retinal pathologies such as age-related macular degeneration, the loss of melanin in RPE was associated with increased inflammation and lipid peroxidization [39]. However, the reliability of evidence is limited, and the role of pigmentation in neonatal retinal physiology has not been clearly established.

Another potential explanation involves genetic differences in pathways regulating angiogenesis and vascular development, including VEGF signaling, HIF, IGF-1, Wnt pathways [40–42]. Variations in these genes could potentially influence susceptibility to oxygen-induced vascular injury. However, few studies have examined genetic variation in ROP risk, with one genome-wide association study identifying certain variants of the Glioma-associated oncogene family zinc finger 3 (GLI3) gene to be significantly associated with stage 3 ROP or worse [43].

These studies are limited and have not identified alleles whose distribution and effect sizes account for the epidemiologic patterns seen in Black and White infants. Hypoxia-induced reactive oxygen species and angiogenic signaling are central to ROP, yet no study has linked race-specific oxidative-stress mechanisms to differential disease risk.

### Socioeconomic determinants of ROP risk

Beyond individual-level factors, structural determinants of health strongly influence prematurity, which is a well-established risk factor for ROP. Neighborhood deprivation indices such as the Area Deprivation Index (ADI) incorporate measures of poverty, education, employment, housing quality, and access to transportation. Higher ADI (indicating greater neighborhood disadvantage) has been associated with missed initial ROP follow-up (up to 30% lower exam completion) [44], which may lead to under-detection of progression to treatment-requiring disease. Similarly, a study using the Child Opportunity Index (COI) found that infants from very low-opportunity neighborhoods had higher odds of treatment-warranted ROP, although the association was attenuated after adjusting for lower GA and BW (indicators of prematurity) [45].

Racial differences often correlate with disparities in socioeconomic status, access to prenatal care, maternal health, and healthcare resources [46]. Black race is strongly associated with high ADI neighborhoods and greater socioeconomic disadvantage, where preterm birth rates increase two-fold due to maternal hypertension, infection, psychosocial stress, and limited prenatal care [47–49]. Furthermore, Black infants are more frequently treated in NICUs with limited resources and healthcare worker understaffing [46, 50–52]. In addition, Black and Hispanic families tend to be more affected by housing instability, food insecurity, and other adverse disparities in mortality and childhood health [53]. On a mechanistic basis, predominantly human breast milk feeding has been associated with improved IGF-1 signaling and lower ROP risk at any stage for infants under 1800 g [12]. Disparities in access to maternal leave, lactation support, and breastfeeding resources may plausibly contribute to variation in IGF-1 mediated vascular development [54, 55]. These additional mechanisms may confound the relationship between race and ROP, including differences in oxygen exposure and survival-related selection.

### Oxygen exposure as a mediating factor

Given the central role of oxygen in ROP pathogenesis, differential oxygen exposure represents a potential mediator linking race and disease risk. Large clinical trials examining oxygen targets have demonstrated that higher oxygen saturation targets were strongly associated with increased rates of treatment-warranted ROP (Type 1) [27]. Oxygen saturation levels in the NICU fluctuate due to variations in clinical practice, respiratory support equipment, and infant physiology.

Recent work has therefore begun to explore alternative explanations involving differences in clinical monitoring and care delivery. For example, racial bias in pulse oximetry has been reported, with pulse oximeters tending to overestimate arterial oxygen saturation in individuals with darker skin pigmentation by 2.4 fold compared to White infants at equivalent true saturations [46, 56]. Survival-related analysis of hospitalized adults with COVID-19 similarly demonstrate that occult hypoxemia (SaO_2_<88% with concurrent SpO_2_ of 92–96%) due to pulse oximeter inaccuracy is more common in Black patients and is associated with reduced access to timely oxygen therapy [57]. Among those who did meet treatment eligibility recognition, Black patients had a median delay of 1 h longer than White patients. Oxygen-targeting trials including SUPPORT, BOOST, and COT demonstrate that relatively small differences in oxygen saturation ranges can alter the incidence of ROP, with lower targets reducing treatment-warranted disease [26–28]. Pulse oximeter overestimation of oxygen saturation in infants with darker skin pigmentation could result in lower true arterial oxygen exposure despite similar SpO_2_ targets. Reduced hyperoxia may thus attenuate phase I vascular suppression and decrease treatment-warranted ROP [32], providing an explanation for lower observed rates among Black infants. However, occult hypoxemia could also disproportionately increase mortality and organ dysfunction, as seen in studies that analyzed clinical outcomes in adults with “hidden hypoxemia” [32, 58]. Death prior to ROP screening may produce a healthy survivor effect, creating the appearance of lower ROP incidence despite higher underlying risk. Thus, these competing factors suggest that clinical studies should consider composite outcomes of death and treatment-warranted ROP [59].

### Survival bias and competing risk

Survival bias, or competing risk bias, may contribute further to the observed racial differences. While screening for ROP begins at 31–34 weeks PMA, treatment-warranted ROP typically develops between 36 and 37 weeks PMA [60]. Infants who die earlier, particularly those born at the lowest gestational ages, may therefore not survive long enough to enter the period of highest risk for ROP. Black infants in the U.S. experience over twice the mortality rate of white infants, experiencing nearly 4-fold as many deaths related to low GA and BW [61]. Methodologic work on racial health disparities has shown that when groups experience different early mortality, racial comparisons are made among selectively healthier survivors rather than the original population at risk (“healthy survivor” selection) [62]. As a result, the remaining cohort of surviving infants may be more clinically stable, potentially lowering the observed incidence of treatment-warranted ROP. Of note, mortality rates were similar across racial groups in the G-ROP-1 cohort, but mainly included infants with incomplete ROP data (infants died before ROP outcome was known, or other reasons such as transfer of care) [11].

A similar phenomenon has been described in BPD risk prediction models, in which increasing respiratory illness severity paradoxically lowered the estimated risk of moderate to severe BPD because the sickest infants die before reaching the diagnostic time point [63]. This illustrates how early mortality can remove the highest-risk infants from the population at risk for later morbidity, thereby distorting observed disease progression. However, most ROP studies do not include racial differences in mortality rates and represents an area for further investigation.

## Discussion

The apparent paradox that Black infants from high-deprivation areas experience greater prematurity yet lower rates of treatment-warranted ROP may reflect survivor bias, differences in follow-up, and variation in clinical monitoring rather than inherent biologic protection. Genetic susceptibility remains a possible contributor, although current evidence is limited and largely speculative. The differential mortality rates between Black and White infants may result in a surviving cohort of Black infants that are relatively more clinically stable at the time when treatment-warranted ROP develops, thereby lowering the observed incidence of treatment-warranted disease. Socioeconomic determinants, including neighborhood deprivation and access to prenatal/postnatal care, also likely play a role in determining ROP risk.

Conflicting findings across studies may reflect differences in oxygen-targeting practices and heterogeneity in study design. Studies like CRYO-ROP, G-ROP, and e-ROP reported neonatal race and ethnicity based on the maternal self-reported race and ethnicity (G-ROP and e-ROP) [4, 11, 16]. In Tanenbaum et al., race and ethnicity were analyzed as a combined variable (race/ethnicity), including non-Hispanic White, non-Hispanic Black, Hispanic or Latino, or other [44]. Notably, this study indicated that mothers will likely be able to identify the race and ethnicity of their child independently at their institution, which may limit inaccurate assumptions based on the solely the mother’s records.

These mechanisms suggest that the apparent protective association between Black race and treatment-warranted ROP may reflect differences in survival patterns, healthcare access, monitoring technology, and clinical management. Future studies integrating accurate oxygen targeting, detailed demographic and socioeconomic reporting, and analytical frameworks that account for competing mortality risks will be essential to clarify these relationships and inform neonatal care.
